# Uganda Public Health Fellowship Program’s Contributions to Malaria Control Programs 2015–2022: Strategies, Implementation Challenges, and Opportunities

**DOI:** 10.9745/GHSP-D-23-00257

**Published:** 2025-12-31

**Authors:** Alex R. Ario, Andrew Kwiringira, Richard Migisha, Benon Kwesiga, Lilian Bulage, Daniel Kadobera, Esther Kisaakye, Alice Asio, Maria’ G. Zalwango, Jane F. Zalwango, Damian Rutazaana, Jimmy Opigo, Julie R. Harris, Kyree Rollins, Mame Niang, Amy L. Boore, Lisa J. Nelson, Kassahun Belay

**Affiliations:** a Uganda Public Health Fellowship Program, Kampala, Uganda.; b Uganda National Institute of Public Health, Kampala, Uganda.; c Uganda National Malaria Control Division, Ministry of Health, Kampala, Uganda.; d U.S. Centers for Disease Control and Prevention, Kampala, Uganda.; e Formerly with the U.S. Agency for International Development, Kampala, Uganda.

## Abstract

The Uganda Public Health Fellowship Program (UPHFP) is a 2-year, non-degree-granting field epidemiology training program. It enrolls only post-Master’s degree fellows, who are integrated during their training into key Ministry of Health (MOH) programs, such as the National Malaria Control Program, and supported technically and financially by the U.S. President’s Malaria Initiative (PMI) and U.S. Centers for Disease Control and Prevention. However, the nature and extent of the UPHFP contributions to the malaria control programs have not been systematically documented. We describe how the UPHFP strategies contributed to malaria control programs and share implementation challenges and opportunities to inform future programming. From 2015 to 2022, UPHFP led or supported 50 malaria projects, including 14 malaria surveillance projects, 11 malaria outbreak investigations, 7 epidemiological studies, 5 case studies, 6 malaria quality improvement projects, 3 policy briefs, and 4 training and mentorship projects. These projects have informed policy decisions and strengthened surveillance, coordination, and response to malaria outbreaks. A key challenge is single-source funding that makes the program more vulnerable to changes in donor priorities. Our documentation demonstrates the critical value of UPHFP to the country’s malaria control efforts by enhancing epidemiologic workforce capacity and strengthening epidemiological surveillance.

## Key Messages

- The Uganda Public Health Fellowship Program (UPHFP) contributed to 50 malaria**-**related projects, including outbreak investigations, surveillance evaluations, and quality improvement initiatives. The UPHFP program also improved malaria surveillance systems by introducing the Malaria Epidemic Early Detection System, enabling the early detection and timely response to malaria outbreaks.
- Embedding fellows into existing Ministry of Health programs enhances workforce capacity. Fellowship programs like UPHFP can serve as a scalable model for other low-resource settings, emphasizing the importance of targeted capacity-strengthening, mentorship, and integration within national health systems.

## BACKGROUND

Over the past 20 years, the scale up of malaria control efforts globally has led to marked reductions in malaria-related morbidity and mortality.^1^ Between 2000 and 2020, an estimated 1.7 billion malaria cases and 10.6 million malaria deaths were averted worldwide.^2^ Sub-Saharan Africa accounted for 95% of the world’s cases in 2020, and, among nations, Uganda contributed 5.1% of the global malaria case burden. Uganda also experienced a 2% rise in malaria incidence between 2021 and 2022, reaching 267.8 cases per 1,000 population at risk.

Uganda has made progress in implementing key malaria control interventions, particularly the distribution of long-lasting insecticide-treated bed nets, indoor residual spraying (IRS) of insecticides, utilization of artemisinin-based combination therapy to treat uncomplicated malaria, and provision of intermittent preventive therapy for pregnant women.^3^ Public health human resource capacity-strengthening programs such as the Uganda Public Health Fellowship Program (UPHFP) have enabled the implementation and evaluation of public health interventions, including malaria control interventions.^4^ However, the nature and extent of UPHFP contributions to malaria control programs have not been systematically documented. We describe how UPHFP strategies contributed to malaria control programs while also sharing implementation challenges and opportunities to inform future programming.

## UGANDA PUBLIC HEALTH FELLOWSHIP PROGRAM IMPLEMENTATION, 2015–2022

Housed in the Uganda National Institute of Public Health (UNIPH), the UPHFP is a 2-year, non-degree-granting program and enrolls only post-Master’s degree fellows. It was established in 2015 by the Uganda Ministry of Health (MOH), with the support of key partners, including Makerere University School of Public Health, the U.S. Centers for Disease Control and Prevention (CDC), and the U.S. President’s Malaria Initiative (PMI).^4^

The program enrolls individuals with various backgrounds in public health, including medical doctors, nurses, and laboratory scientists. Some fellows are in government service while others come from various sectors, including academia, nongovernmental organizations, or health care facilities. The fellows are selected through a competitive application and interview process. Selection committees evaluate these applications based on specific criteria, including academic qualifications, relevant experience, and commitment to public health.

The fellows receive monthly stipends intended to cover living expenses and enable them to focus on their professional development. They are paired with experienced mentors who provide guidance, supervision, and support throughout the fellowship period. These mentors include UPHFP program employees, the U.S CDC resident advisor, and, formerly, USAID Resident PMI Advisors.

During the 2-year fellowship period, UPHFP fellows are integrated into key MOH programs (host sites), such as the National Malaria Control Program, AIDS Control Program, National Tuberculosis and Leprosy Control Program, Maternal and Child Health Department, Integrated Epidemiology, Surveillance, and Public Health Emergencies department, Vaccines and Immunization Division, and the Emergency Operations Center. In their host sites and during mentored field projects, fellows gain competencies in 6 domains^4^:

- Conducting outbreak investigations
- Conducting applied epidemiologic studies
- Analyzing surveillance data and evaluating a surveillance system
- Conducting economic evaluations
- Preparing policy briefs that distill research findings in plain language and draw clear links to policy initiatives and quality improvement projects
- Management and leadership

After completing their fellowships, the fellows often have several career pathways: Some continue to work within government agencies, applying their newly acquired skills and expertise to public health programs and initiatives, while others pursue careers in academia, nongovernmental organizations, or international agencies. Career choices vary based on their individual interests and opportunities.

## UPHFP CONTRIBUTIONS TO MALARIA CONTROL IN UGANDA

Between 2015 and 2022, 15 UPHFP fellows were supported by PMI. During these 7 years of program implementation, the fellows completed 50 applied epidemiology malaria projects. Of these projects, 11 were malaria outbreak investigations (Figure) while 14 were malaria surveillance projects, including analysis of malaria surveillance data or evaluation of malaria surveillance systems, 7 were epidemiological studies, 5 were case studies, and 6 were malaria quality improvement projects (Table 1). The fellows also synthesized evidence from these projects and developed policy briefs and published peer-reviewed articles to contribute to the growth of the knowledge base.

**FIGURE:**
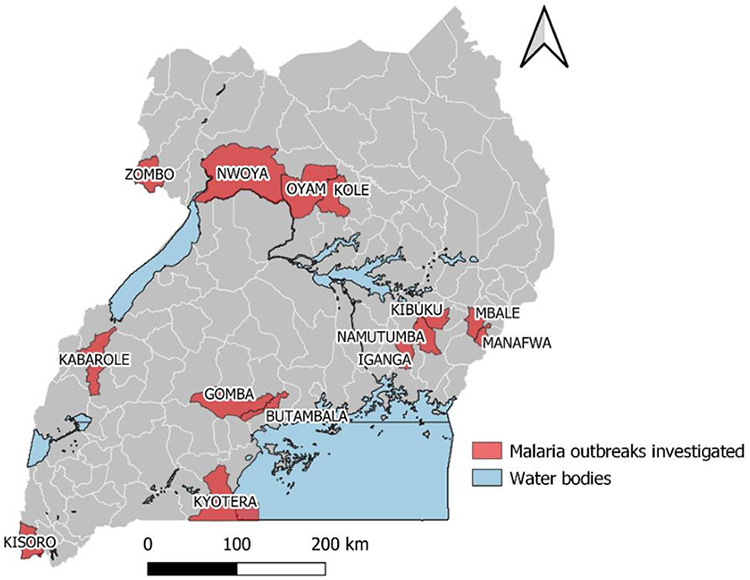
Distribution of Malaria Outbreaks Investigated by Uganda Public Health Fellowship Program Fellows, Uganda, 2015–2022

**TABLE 1.** Contributions of the Malaria Fellows of the Uganda Public Health Fellowship Program to Malaria Control in Uganda, 2015–2022

**Activities**	**N**
**Malaria Control and Prevention Projects**	
Evaluation of surveillance systems	13
Outbreak investigations	11
Quality improvement projects	7
Epidemiological studies	6
Case studies	6
Policy briefs	3
Training and mentorship	4
**Malaria-Related Scientific Publications**	
Peer-reviewed, published, or accepted	7
Published in UNIPH quarterly bulletin	20
**Malaria Weekly Bulletin**	Published since 2015
**Malaria Quarterly Bulletin**	Published since 2015

Abbreviation: UNIPH, Uganda National Institute of Public Health.

### Coordination of Malaria Epidemic Response

Before UPHFP existed in Uganda, response to epidemics was not well coordinated and outbreak investigations were frequently conducted in administrative silos by different departments of the MOH. Since the commencement of the UPHFP, there have been significant improvements in the coordination and implementation of outbreak investigations and response in Uganda.

UPHFP fellows are members of the National Rapid Response Team during emergencies and outbreak investigations, including malaria outbreak investigations. Outbreak reports prepared by fellows are routinely discussed in the National Public Health Emergency Operation Center and presented at the National Task Force for Epidemic Preparedness and Response, which provides guidance and oversight to disease outbreak prevention and control in the country. For example, in 2021 and 2022, multiple malaria outbreak investigations by fellows informed strategic decisions for the malaria program. A notable example includes the 2018 outbreak report on blackwater fever cases (a severe complication of malaria). This report, combined with follow-up case-control studies, have informed decisions by the National Malaria Control Division of the MOH to: a) update Uganda clinical guidelines by including the diagnosis, clinical presentation, and management of blackwater fever; b) prioritize blackwater fever for emergency response in the malaria strategic response plan; and c) select districts to prioritize for introduction of the malaria vaccine in Uganda.

Although the Government of Uganda has made notable progress in strengthening political commitment to prevent and control public health emergencies, challenges remain in the timely release of domestic funds required for rapid epidemic response, including malaria outbreaks. Since its inception, UPHFP, supported by the US Government, has helped bridge these gaps by enabling rapid access to resources and deployment to affected areas during emergencies. However, under the current America First Global Health Strategy, which reorients US global health assistance toward multiyear bilateral agreements focused on strengthening local health systems, reducing inefficiencies, and promoting sustainability, the mechanisms for disbursing funds to UNIPH will change, although the overall funding level is not expected to be significantly affected in the short term.

### Malaria Surveillance and Early Warning System

Before UPHFP, the MOH did not have an active malaria epidemic alert system to aid the detection of malaria outbreaks. In 2016, UPHFP fellows created such a system to monitor malaria “normal channels” (normal thresholds) using existing data in real time and identify upsurges as soon as they occur. Early detection is crucial for containing outbreaks before they escalate into larger public health crises. To confirm the existence of a malaria upsurge at an early stage, the fellows compared weekly malaria cases to the expected number of cases for a particular district during a similar period of the year based on data from the previous 5 complete years. The system created by the UPHFP fellows allowed for the early detection of 14 malaria outbreaks until 2022 and enabled the team to respond and initiate timely preventive and/or control measures.

On a weekly basis, UPHFP fellows review and analyze malaria surveillance data to assess the malaria situation, the performance of the surveillance system, and to guide necessary response(s). The fellows also create feedback mechanisms from the central to lower levels of the health system by generating a weekly malaria bulletin (Table 2). The bulletin has been running regularly since 2015, with fellows taking the lead in its production. The bulletin has enabled the MOH and its partners to devise strategies to address obstacles to malaria control. For example, a recent evaluation of malaria epidemic alert thresholds (“malaria normal channels”) has informed redefining what constitutes malaria epidemics, and a report on risk factors for deaths among children with severe malaria in the district of Namutumba informed strengthening of community malaria death surveillance. More so, the bulletin is disseminated to district health officers who use the report to monitor stock levels of essential supplies like antimalarials, ensuring timely redistribution to areas with shortages.

**TABLE 2.** Malaria Projects Implemented by the Uganda Public Health Fellowship Program, 2015–2022

**Year**	**Project Titles**
**Outbreak Investigations**	
2018	Malaria Outbreak Investigation in Kisoro District, January 2018
2018	Malaria Outbreak Investigation in Nwoya District, April 2018
2018	Suspected Black Water Fever among Children in Manafwa District, Eastern Uganda, 2018
2019	Malaria Outbreak Facilitated by Increased Mosquito Breeding Sites near Homes and Cessation of Indoor Residual Spraying, Kole District, January–June 2019
2019	Malaria Outbreak Facilitated by Roadside Pools in Zombo District, Uganda, January–June 2019
2019	Malaria Outbreak Facilitated by Increased Vector-breeding Sites Sustained by Intermittent Rainfall: Mbale District, Uganda
2019	Malaria Outbreak Propagated by Rain Water Harvesting and Increased Vector Bleeding Sites, Butambala District, Uganda, August 2018–February 2019
2020	Malaria Outbreak Facilitated by Appearance of Vector-Breeding Sites after Heavy Rainfall and Inadequate Preventive Measures: Nwoya District, Northern Uganda, February–May 2018
2021	Malaria Outbreak Facilitated by Agricultural Activities, Residing near Water-logged Areas and Participating in Late Night Campaign Activities: Nabitende Subcounty, Iganga District, December 2021–February 2021
2021	Suspected Black Water Fever among Children in Districts of Bugisu, Bukedi, and Busoga Region, Eastern Uganda, January 2019–July 2021
2015	Decline in Malaria Incidence in Tororo District, Uganda Following Implementation of Vector Control Interventions, 2013–2015
**Analysis of Surveillance Data**	
2015	Malaria Morbidity among Children under 5year in Epidemic Districts of Northern Uganda for the Period July 2012 to June 2015
2022	Investigation of Malaria Upsurge in Indoor Residual Spraying (IRS) Districts in Eastern Uganda
2016	Factors Associated with Uptake of Optimal Doses of Intermittent Preventive Therapy for Malaria among Pregnant Women in Uganda: Analysis of Data from the Uganda Demographic and Health Survey, 2016
2016	Effect of Active Testing and Treatment on Community Malaria Parasite Prevalence in High Transmission Villages in Tororo District
2016	Establishing Entomological Surveillance in Uganda
2016	Rapid Reduction of Malaria Following Introduction of Vector Control Interventions in Tororo District, Uganda: A Descriptive Study
2017	Malaria Incidence among Children less than 5 years During and after Cessation of Indoor Residual Spraying in Northern Uganda
2017	Malaria Morbidity Following Indoor Residual Spraying in Eastern and Northern Uganda: A Comparative Analysis of IRS and non-IRS districts 2013–2016
2017	Spatial Analysis of Insecticide Resistance among Malaria Vectors in Uganda
2018	Assessing Morbidity and Mortality Patterns of Malaria in Pregnancy using the Health Management Information System (HMIS) in Uganda, July 2014 to June 2017
2019	Evaluation of Community-level Malaria Reporting by Village Health Teams, Uganda, 2018-2019
2021	Effect of Seasonal Malaria Chemoprevention on Incidence of Uncomplicated Malaria among Under 5 Children in Kotido and Moroto Districts Uganda 2021
2021	Evaluation of Indoor Residual Spraying (IRS) in IRS districts, Uganda, 2021
2021	Malaria Outbreak Facilitated by Engagement in Activities near Swamps Following Increased Rainfall and Limited Preventive Measures: Oyam District, Uganda
**Epidemiological Studies**	
2018	Investigation of Increased Deaths due to Severe Malaria in Hoima and Kabarole Districts, 2017–2018
2019	Assessment of Health Workers Adherence to the Malaria Test Treat and Track Policy in Uganda, 2019-2020
2020	Integrated Community Case Management (ICCM) and Antenatal Care-Baseline Survey, 2020
2021	Ownership and Use of Long-lasting Insecticidal Nets and Factors Associated, Immediately after a Mass Distribution Campaign in Uganda: A Cross-sectional Survey of Fourteen districts
2021	Investigation of Risk Factors Associated with Malaria Deaths in Agago District in Uganda in the Period January 2020 to date, May 2021
2021	Investigation of Risk Factors for Death among Children with Severe Malaria in Namutumba District, Eastern Uganda, Sept 2021–Feb 2022
2022	You Can’t Find What You Are Not Looking For: Undetected Malaria Outbreaks Detected through Analysis of Surveillance Data
**Case Studies**	
2022	Malaria Outbreak in Mbale: It’s the Pits!
2022	Delayed Malaria Outbreak Detection: A Wake-Up Call to Evaluate the Malaria Surveillance System
2022	Preparing for the Worst: Opportunities to Prevent Trans-boundary Disease Transmission in Uganda
2022	Investigating a Malaria Outbreak: Case Study
2022	An Outbreak of Malaria Caused by Engagement in Commercial Activities in Swamps
**Policy Briefs**	
2018	Combating Resistance to Insecticides: Alternative Approaches in Sustaining the Relevance of Vector Control Strategies
2020	The Magic Bullet: Using Interpersonal Communication to Increase Consistent Bed Net Use in Uganda
2018	Improving Malaria Reporting by Village Health Teams under Integrated Community Case Management
**Quality Improvement**	
2018	Improving Adherence to Malaria Test and Treat Policy in Iganga Hospital
2021	Improving Malaria Mortality Audits in Hoima Regional Referral Hospital, January–July 2022
2021	Quality Improvement on Early Identification and Management of Malaria Cases to Reduce on Deaths in Bugono Health Center IV, Iganga District
2021	Improving Access to Integrated Community Case Management (ICCM) Services for Malaria in the Community
2022	Mentor Mothers can Improve Malaria in Pregnancy Outcomes
2022	Improvement of Reporting Malaria Death Data via the District Health Information Software 2 in Selected Districts in Uganda
**Training and Mentorship**	
2021	Training on Normal Malaria Channels in Multiple Districts
2020	Training District Health Teams (DHTs) on Developing and Interpreting Malaria Normal Channels to Detect Malaria Outbreaks in Uganda
2021	Continuous Training of District Health Teams through the Frontline Field Epidemiology Training Program on Disease Surveillance and Outbreak Investigation
2022	Mentorship of Health Workers on Managing Malaria in Pregnancy

Several impact evaluation projects have been conducted by fellows to inform policy (Table 2), including a study that assessed the impact of IRS on the incidence of malaria among children under 5 years old.^7^ This study showed that, following IRS in November and December 2015, malaria incidence dropped for 6 months, after which it started increasing until an epidemic was declared in June 2015, right after a peak in rainfall. Authors of the study recommended that IRS be rescheduled to coincide with the build-up of vector populations just before the onset of the peak transmission season, rather than introducing it anytime when IRS insecticides become available. Since then, the recommendations have been adopted.

Other studies conducted by the fellows have provided similarly valuable insights. For example, investigations into malaria outbreaks driven by rainwater harvesting and roadside pools revealed the role of environmental factors in sustaining mosquito breeding. These findings informed recommendations for targeted vector control interventions, such as reducing breeding sites in addition to conventional tools like insecticide-treated nets and IRS. Furthermore, the rainwater harvesting study led to community-level educational initiatives to mitigate the unintended consequences of water storage practices, demonstrating the program’s ability to influence both policy and grassroots-level behavior change.

### Teaching and Mentoring

UPHFP fellows trained and developed the capacity of District Health Teams (DHTs) in activities related to their expected tasks (Table 2), including analyzing surveillance data and developing visualizations to improve malaria data use. Other capacity-building activities included training DHTs in epidemic-prone districts on how to establish the Malaria Epidemic Early Detection System (MEDS) for timely detection of malaria epidemics. The MEDS uses malaria surveillance data to develop malaria epidemic alert thresholds (“malaria normal channels”).^8^ The malaria normal channels help the DHTs to detect outbreaks and deploy timely interventions to prevent or mitigate the scope and impact of epidemics on the population.

UPHFP fellows are also directly involved in the mentorship and training of Field Epidemiology Training Program (FETP)-Frontline trainees (Table 2). The FETP-Frontline is a 3-month in-service training program to strengthen epidemiological capacity at the district level and focuses on the detection of and response to epidemic-prone diseases at the source. Participants come together for 3 classroom workshops; between workshops, FETP-Frontline participants return to their jobs and complete field projects to practice, implement, and reinforce what they have learned. Mentorship of FETP-Frontline trainees is critical in the production, interpretation, and updating of malaria normal channels as well as other surveillance activities.

### Public Health Communications

UPHFP published 8 malaria-related articles in peer-reviewed journals^6^^,^^7^^,^^9^^–^^14^ and 20 articles in the UNIPH quarterly epidemiological bulletin by December 2022 (Table 2). In addition to the manuscripts, PHFP has had several malaria-related abstracts accepted for presentation at national and international conferences.

UPHFP fellows have developed and delivered oral public health communications and conducted risk communication activities to spread awareness and combat misinformation about malaria in Uganda, including engaging communities through social media, radio, press interviews, and public awareness campaigns. Some newspaper articles, such as “Why the fight against malaria should continue in Uganda,”^15^ were published in the national newspaper. The article highlighted the population at risk of poor outcomes of malaria and how the population can prevent malaria.

### Management and Leadership

UPHFP fellows and graduates have held leadership positions in a variety of MOH departments. For example, supervisors and coordinators at the UNIPH as well as program leads at the MOH include several graduates of UPHFP, some of whom continue to provide valuable contributions to malaria control in the country. FETP fellows were also involved in the planning and coordination of activities in the MOH National Malaria Control Program, serving as subject matter experts in risk communication, monitoring and evaluation, statistical analysis, and grant writing. UPHFP fellows participated in the writing of a Global Fund concept note, which enabled the government to obtain funding for the 2024–2027 Global Fund grant for malaria. The concept note writing process is a critical activity led by the MOH, as it secures funding for national health programs, including malaria control efforts. The fellows contributed to key areas, including statistical analysis, monitoring and evaluation, and drafting technical sections of the concept note.

### Quality Improvement and Policy Briefs

According to the WHO framework for quality of care,^16^ health systems should endeavor to improve in 6 essential areas: efficacy, efficiency, accessibility, acceptability, equity, and safety. With a focus on enhancing the quality and effectiveness of health systems in these priority areas, UPHFP fellows carry out quality improvement projects (Table 2). One such project on the malaria treat-and-track policy included mapping gaps within the continuum of care and making recommendations to address the gaps.

UPHFP fellows’ policy papers on malaria have compiled research evidence and provided recommendations on potential courses of action based on knowledge gained from various situations. For instance, a policy brief on combating pesticide resistance (Table 2) summarized evidence for increasing insecticide resistance and emphasized the urgency of using synergist piperonyl butoxide (PBO)-enhanced mosquito nets to combat insecticide resistance. These briefs were specifically targeted at decision-makers within the MOH, the National Malaria Control Program, and implementing partners, with the aim of informing and improving malaria control policies.

## IMPLEMENTATION CHALLENGES

UPHFP has faced several implementation challenges, including reliance on a single funding source, which increases vulnerability due to shifts in donor priorities. While Uganda government political and policy support for public health initiatives has strengthened, the evolving US funding landscape under the America First Global Health Strategy will alter funding disbursement mechanisms to UNIPH, albeit, without significantly affecting overall funding levels in the short term.

## DISCUSSION

The UPHFP has contributed to the malaria control efforts in Uganda. The contributions of the fellows match well with the core competencies of their training: conducting outbreak investigations, applied epidemiologic studies, and economic evaluations, analyzing surveillance data and evaluating a surveillance system, preparing policy briefs, planning quality improvement projects, and managing and leading key MOH programs. The effectiveness of UPHFP fellows implementing key activities of the Uganda malaria reduction strategy can be attributed to the integration of UPHFP into MOH programs.

Malaria programs operate in a complex environment, with a continuous need to adjust responses to outbreaks, changing transmission patterns, and the development of drug and insecticide resistance. This requires significantly expanded human resource capacities at the national, district, and community levels.^15^ The education and training of health workers, program staff, and malaria researchers—including adequate mentoring and supervision—is the key to ensuring a robust dynamic response to malaria challenges. Also, new tools on the horizon for malaria prevention and control, such as next-generation insecticide-treated nets, new chemoprevention regimens, and innovative diagnostic technologies, require new skills for introduction and even further investments in capacity strengthening. These dynamics underscore the value of UPHFP in building a field epidemiology workforce that is critical to handling the ever-changing public health malaria priorities. Programs similar to UPHFP are increasingly acknowledged as effective pathways for enhancing human resource capacities.^16^

Ethiopia’s FETP and Nigeria’s National Malaria Elimination Program have served as influential models in shaping Uganda’s surveillance systems. Ethiopia’s FETP demonstrated the effectiveness of embedding epidemiology fellows within district health offices to address critical gaps in disease reporting and outbreak response. This approach provided valuable insights for Uganda’s integration of fellows into district and national malaria programs, thereby enhancing surveillance capabilities and improving outbreak management. Similarly, Nigeria’s National Malaria Elimination Program emphasized the integration of malaria training with routine health data systems. This strategy guided Uganda’s efforts to strengthen the quality and utilization of malaria case management data, facilitating more accurate outbreak detection and timely responses.

UPHFP offers valuable lessons for similar programs in other countries. Uganda’s approach to embedding fellows within both national and subnational health structures ensures their skills are utilized in decision-making and implementation across multiple levels. The program’s focus on reintegration of fellows into permanent roles and emphasis on producing actionable outputs like surveillance bulletins and outbreak reports provides a model for addressing specific public health challenges while ensuring sustainability. Additionally, Uganda’s emphasis on multisectoral collaboration demonstrates the importance of aligning fellowship activities with national health priorities, offering a replicable framework for building resilient public health systems.

UPHFP has made significant strides in tackling Uganda’s public health issues. To enhance institutionalization and sustainability, the program could explore diversified funding sources, engage with local partners, and advocate for continued government support. Strengthening collaboration with the MOH and other government agencies is also crucial to sustainability. The program could also explore opportunities for knowledge sharing and collaboration with regional and international partners. This collaboration could expose the program to a wider variety of public health experiences, approaches, and best practices. This exposure would help inform program design and foster innovation and adaptation to Uganda’s dynamic public health needs.

## CONCLUSION

The UPHFP program has contributed to capacity strengthening, more systematic and strategic collection of surveillance data, and improvements to the National Malaria Control Program’s deployment of malaria control strategies. To sustain these gains, the government, donors, and stakeholders should continue to support the program. Uganda’s model of embedding fellows into existing MOH programs showcases the importance of leveraging existing structures to maximize impact. The program focused on developing key competencies such as outbreak investigations, surveillance analysis, and policy development that are critical for tackling dynamic health challenges like malaria. This targeted skill-building addresses gaps in workforce capacity that many countries struggle with, particularly in sub-Saharan Africa.

## Notes

**Peer Reviewed**
